# Characterization of a Lineage C.36 SARS-CoV-2 Isolate with Reduced Susceptibility to Neutralization Circulating in Lombardy, Italy

**DOI:** 10.3390/v13081514

**Published:** 2021-07-31

**Authors:** Matteo Castelli, Andreina Baj, Elena Criscuolo, Roberto Ferrarese, Roberta A. Diotti, Michela Sampaolo, Federica Novazzi, Daniela Dalla Gasperina, Daniele Focosi, Davide Ferrari, Massimo Locatelli, Massimo Clementi, Nicola Clementi, Fabrizio Maggi, Nicasio Mancini

**Affiliations:** 1 Laboratory of Microbiology and Virology, Vita-Salute San Raffaele University, 20132 Milan, Italy; castelli.matteo@hsr.it (M.C.); criscuolo.elena@hsr.it (E.C.); ferrarese.roberto@hsr.it (R.F.); diotti.robertaantonia@hsr.it (R.A.D.); clementi.massimo@hsr.it (M.C.); clementi.nicola@hsr.it (N.C.); 2 Laboratory of Microbiology, ASST Sette Laghi, 21100 Varese, Italy; andreina.baj@uninsubria.it (A.B.); federica.novazzi@asst-settelaghi.it (F.N.); 3 Department of Medicine and Surgery, University of Insubria, 21100 Varese, Italy; d.dallagasperina@uninsubria.it; 4 Laboratory of Microbiology and Virology, IRCCS Ospedale San Raffaele, 20132 Milan, Italy; sampaolo.michela@hsr.it; 5 Internal Medicine Unit, ASST Sette Laghi, 21100 Varese, Italy; 6 North-Western Tuscany Blood Bank, Pisa University Hospital, 56127 Pisa, Italy; d.focosi@ao-pisa.toscana.it; 7 SCVSA Department, University of Parma, 43121 Parma, Italy; davide.ferrari@unipr.it; 8 Laboratory Medicine Service, IRCCS San Raffaele Scientific Institute, 20132 Milan, Italy; locatelli.massimo@hsr.it

**Keywords:** SARS-CoV-2, COVID-19, variant of concern, L452R, R346K, spike

## Abstract

SARS-CoV-2 spike is evolving to maximize transmissibility and evade the humoral response. The massive genomic sequencing of SARS-CoV-2 isolates has led to the identification of single-point mutations and deletions, often having the recurrence of hotspots, associated with advantageous phenotypes. We report the isolation and molecular characterization of a SARS-CoV-2 strain, belonging to a lineage (C.36) not previously associated with concerning traits, which shows decreased susceptibility to vaccine sera neutralization.

## 1. Introduction

SARS-CoV-2 evolution is leading to the accumulation of single-point mutations and deletions in the spike gene. The majority of them can be attributed to genetic drift as they do not correlate with a clear selective advantage. However, a few spike variants are associated with increased fitness and/or reduced neutralization, as demonstrated by their high incidence and recurrence in phylogenetically unrelated SARS-CoV-2 isolates. The Center for Disease Control and Prevention currently defines SARS-CoV-2 variants of interest (VOI) those carrying one or more of these genetic markers, and variants of concern (VOC) those having a clear selective advantage at the whole-virus level. Here we describe the first molecular characterization of a lineage C.36 isolate (named hCoV-19/Italy/LOM-UnINSU/2021) carrying spike mutations associated with multiple VOCs. At the time of isolation, this spike configuration had been documented just once in Thailand; however, since then, a steadily increasing number of highly related isolates has been sequenced in Lombardy (Italy), Europe, and the USA. Our experimental and computational analyses suggest that this emerging C.36 sublineage has a reduced susceptibility to neutralization that recommend its close monitoring.

## 2. Materials and Methods

### 2.1. Clinical Samples

Twenty serum samples were collected from COVID-19, Comirnaty^®^ (Pfizer-BioNTech, New York, NY, USA) vaccinated subjects, a month after receiving the second dose. Subjects #1 and #2 had a clinical history of SARS-CoV-2 infection before being vaccinated, while the others did not. Nasopharyngeal swabs were processed on the MDX Platform (DiaSorin, Saluggia, Italy) or the m2000 platform (Abbott, Chicago, IL, USA).

### 2.2. Immunoassays

Elecsys Anti-SARS-CoV-2 Cobas^®^ (Roche Zurich, Switzerland) and LIAISON^®^ XL Analyzer SARS-CoV-2 S1/S2 IgG assay (DiaSorin, Saluggia, Italy) were used for detecting anti-SARS-CoV-2 antibodies in all serum samples. The electrochemiluminescence immunoassay (ECLIA) can detect the presence of IgG, IgM, and IgA antibodies recognizing the S protein (anti-RBD). According to the manufacturer, samples positive for anti-SARS-CoV-2 antibodies show a cutoff index (COI) equal to or greater than 1. All samples with a COI <1.0 are considered negative for the presence of SARS-CoV-2 antibodies. The SARS-CoV-2 S1/S2 IgG assay by DiaSorin can detect IgG antibodies directed against the S protein (S1 and S2). According to the manufacturer instructions, samples featuring <12.0 AU/mL are considered negative, those ranging between 12.0 and 15.0 AU/mL are undetermined, and those above 15 AU/mL are positive.

### 2.3. Virus and Cells

Vero E6 (Vero C1008, clone E6-CRL-1586; ATCC) cells were cultured in Dulbecco’s Modified Eagle Medium (DMEM) supplemented with non-essential amino acids (NEAA), penicillin/streptomycin (P/S), HEPES buffer, and 10% (*v*/*v*) fetal bovine serum (FBS). Four clinical isolates of SARS-CoV-2 were obtained and propagated in Vero E6 cells: D614G (hCoV-19/Italy/UniSR1/2020; GISAID Accession ID: EPI_ISL_413489), B.1.1.7 (Alpha) (19/Italy/LOM-UniSR7/2021; GSAID Accession ID: EPI_ISL_1924880), C.36_3 (hCoV-19/Italy/LOM-UnINSU/2021, GISAID Accession ID: EPI_ISL_1509923), B.1.351 (Beta) (hCoV-19/Italy/LOM-UniSR6/2021, GISAID Accession ID: EPI_ISL_1599180).

In detail, 0.8 mL of the transport medium of the nasopharyngeal swab (COPAN’s kit UTM^®^ universal viral transport medium—COPAN) was mixed 1:1 with DMEM without FBS, and supplemented with P/S and Amphotericin B. The mixture was added to 80% confluent Vero E6 cells monolayer seeded into a 25 cm^2^ tissue culture flask. After 1 h adsorption at 37 °C, 3 mL of DMEM supplemented with 2% FBS and Amphotericin B were added. One day post-infection (dpi), the monolayer was washed in PBS and 4 mL of DMEM supplemented with 2% FBS and Amphotericin B were added. The cytopathic effect was monitored in inverted phase-contrast microscopy (Olympus CKX41) and the supernatant was collected at monolayer complete disruption (3 dpi). The sample was heat-inactivated at 56 °C for 30 min and the viral genome was extracted using QIAamp Viral RNA Mini Kit following the manufacturers’ instructions. Extracted RNA was processed with the CleanPlex^®^ SARS-CoV-2 Panel (Paragon Genomics, Hayward, CA, USA) and sequenced with MiSeq Reagent Kit v2 (300-cycles) (Illumina, San Diego, CA, USA) on the MiSeq platform. Genomic reconstruction was performed using the SOPHiA DDM™ platform (SOPHiA Genetics, Lausanne, Switzerland).

### 2.4. Virus Titration

Virus stocks were titrated using Endpoint Dilutions Assay (EDA, TCID50/mL). Vero E6 cells were seeded into 96-well plates and infected at 95% of confluency with base 10 dilutions of virus stock. After 1 h of adsorption at 37 °C, the cell-free virus was removed, cells were washed with PBS 1×, and complete medium was added to cells. After 72 h, cells were observed to evaluate the presence of a cytopathic effect (CPE). TCID50/mL of viral stocks were then determined with the Reed–Muench formula.

### 2.5. Microneutralization Experiments

Vero E6 cells were seeded into 96-well plates 24 h before the experiment, performed at 95% cell confluency for each well. Serum samples were decomplemented by incubation at 56 °C for 30 min, and two dilutions (1:80 and 1:160) were incubated with SARS-CoV-2 at 0.01 multiplicity of infection (MOI) for 1 h at 37 °C. Virus–serum mixtures and positive infection control were applied to Vero E6 monolayers after washing cells with PBS 1×, and virus adsorption was carried out at 37 °C for 1 h. Then, the cells were washed with PBS 1× to remove cell-free virus particles and virus-containing mixtures and cultured in complete DMEM supplemented with 2% FBS. The plates were incubated at 37 °C in the presence of CO_2_ for 72 h. The experiments were performed in triplicate. Neutralization activity was evaluated by comparing CPE presence detected in the presence of virus–serum mixtures to positive infection control.

### 2.6. In Silico Analysis

The ectodomain structure (residue 14–1146) of hCoV-19/Italy/LOM-UnINSU/2021 was modeled with MODELLER using the cryo-EM structure of SARS-CoV-2 D614G spike (RCSB ID: 7BNN) as a template. The RBD-ACE2 complex (residue 19–614 and 334–527, respectively) was modeled with MODELLER using the structure with RCSB ID: 6M17. All structures were simulated in an orthorhombic TIP3P water box, neutralized with the proper counterions, and parametrized using the all-atom AMBER/parm12SB force field [1]. All simulations were performed using the GROMACS 5.1.4 code [2]. Periodic boundary conditions in the three axes were applied. Covalent bond length, including hydrogen bonds, was set using the LINCS algorithm, allowing a time-integration step of 2 fs. Constant pressure was imposed using the Parrinello–Rahman barostat with a time constant of 2 ps and a reference pressure of 1 bar, while the constant temperature was maintained using the modified Berendsen thermostat with a time constant of 0.1 ps. Long-range electrostatic interactions were calculated with the particle mesh Ewald method with a real-space cutoff of 12 Å. Each system was minimized with the steepest descent algorithm, equilibrated for 100 ps in an NVT ensemble followed by 100 ps in an NPT ensemble, and then subjected to a 200 ns simulation at constant temperature (300 K). Spike trajectories were analyzed in VMD using the dihedrals described by Henderson et al. using in-house implemented scripts [3]. The cryo-EM structures of SARS-CoV-2 spike in the 1-up and closed conformation were used as reference (RCSB ID: 6ZGG and 6ZGI, respectively). For affinity calculations, the wild type and double mutant RBD-ACE2 complexes trajectories were restarted at the end of the 200 ns trajectories to produce 10 independent runs for each system (10 ns each). Affinity was calculated using MMGBSA corrected as in [4].

## 3. Results and Discussion

A 79-year-old female host of a nursing home suffering from systemic arterial hypertension, gout, and stage 4 chronic renal failure, was admitted to the Varese hospital emergency room on 20 February, 2021, for desaturation. She tested positive for SARS-CoV-2 on the nasopharyngeal swab (NPS) (cycle threshold (Ct) 16 on MDX Platform) and was initially treated with low-molecular-weight heparin, prednisone, and azithromycin. Anti-SARS-CoV-2 S1/S2 IgG in serum was negative with DiaSorin Liaison^®^, suggesting a primary infection. She was then moved to the COVID hub of the hospital, and finally to the hospice ward on 1 March. NPS was positive on 28 February (Ct 6.5 on m2000 platformA), and 1 March (Ct 7.6 on m2000), evidencing an increase in viral load. The clinical course was complicated by urosepsis, required reservoir mask ventilation, and the patient finally died on 4 March.

**Figure 1:**
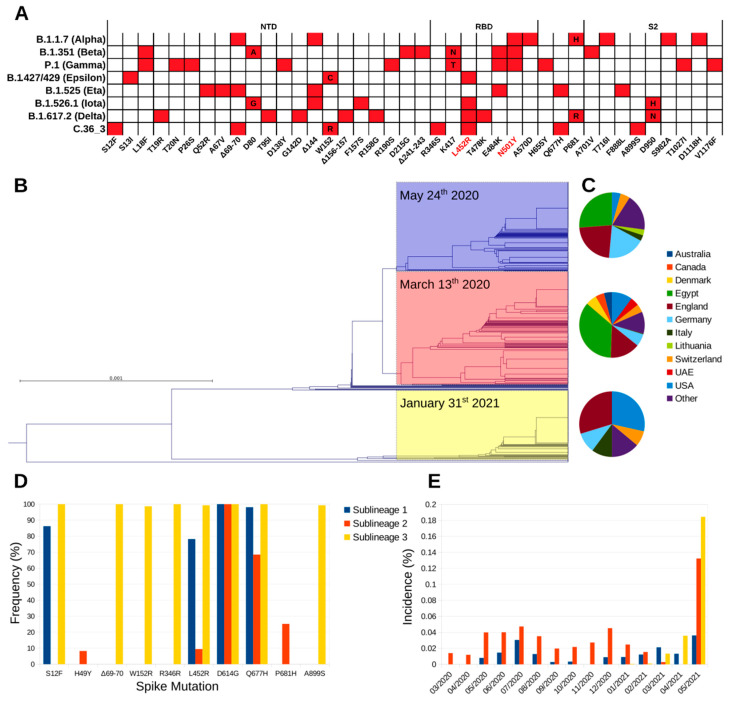
Phylogeny and distribution of lineage C.36. (**A**) Spike amino acid mutations associated with the currently most relevant VOC and VOI. Mutations associated with increased affinity are highlighted in red. (**B**) Phylogram of C.36 spike amino acid sequences deposited on GISAID before 18 May 2021, with sublineage 1, 2, and 3 highlighted in blue, red, and yellow, respectively. For each sublineage, the first sampling date is reported. (**C**) Geographic distribution of the three sublineages. (**D**) Spike amino acid mutations most frequently associated to each C.36 sublineage. (**E**) Sublineages global monthly incidence normalized by the total number of sequenced isolates over the same period.

Illumina™ whole-genome sequencing (WGS) on the first NPS identified the strain (hCoV-19/Italy/LOM-UnINSU/2021, hereafter named C.36_3) as belonging to clade 20D and lineage C.36 (Nextclade and Pangolin definitions as of 18 May 2021, respectively). At the time of sampling, only one nearly identical sequence genome-wide (featuring only four single nucleotide polymorphisms) had been previously reported in Bangkok in a Thai tourist returning from Egypt on 31 January 2021 (isolate hCoV-19/Thailand/CU-SI2104740-NT/2021, EPI_ISL_1007659). Since then, isolates with identical spike sequences were reported mostly in USA and Europe and multiple times in Lombardy, in Northern Italy (Figure 1). The C.36 lineage can be divided in three sublineages according to the spike amino acid sequence (Figure 1B). Worth mentioning, during manuscript revision, Pangolin lineage C.36 was further divided into C.36.1, C.3.6.2, and C.36.3. Isolate C.36_3 described in this study and homologues all fall into lineage C.36.3. Strains belonging to sublineage 1 and 2 were identified first in March 2020, and show comparable geographic distribution, with prevalence being the highest in Egypt, where the relevant sublineage 2 increase in May 2021 was associated with the selection of the spike mutation L452R. The first identification of sublineage 3 is relevantly more recent (late January 2021), and since then has been steadily increasing, albeit the global incidence is still low (Figure 1B,E). Compared to the other C.36 sublineages, it shows a marked difference in geographic distribution, as it is diffused mainly in Europe and USA (Figure 1C and Table S1), and a higher number of spike variations that are conserved among isolates belonging to it (Figure 1A,D). In detail, the identified mutations and deletions are: S12F, ΔH69-V70, W152R, R346S, L452R, D614G, Q677H, and A899S. Of note, VOC B.1.427/B.1.429 (Epsilon) also carries analogous mutations (S13I and W152C) in the N-terminal domain (NTD) that dramatically reduce the neutralizing potency of antibodies specific for this domain [5]. NTD-specific neutralization escape is also well documented for ΔH69-V70 that is found in VOC B.1.1.7 (Alpha) and VOI B.1.525 (Eta). The mutations R346S and L452R lie within the receptor-binding domain (RBD) and the latter has been characterized in SARS-CoV-2 isolates of VOI B.1.427/B.1.429, VOI B.1.526.1 (Iota) and VOI B.1.617.2 (Delta) [6]. Mutation L452R causes resistance to the clinically approved monoclonal antibody (mAb) LY-CoV555 (bamlanivimab) [7] and, in the context of VOC B.1.427/B.1.429 pseudoviruses, to a relevant fraction of other anti-RBD mAbs [5,8,9]. Mutation R346S has a very low global frequency and has been selected in vitro under the selective pressure from class 3 (not directly interfering with ACE2 binding) mAb C135 [10,11]. Finally, the spike is also characterized by the highly prevalent D614G mutation that increases SARS-CoV-2 transmissibility, Q677H that is found in VOI B.1.525, and the rare A899S. Overall, C.36_3 shows a spike configuration similar to VOI B.1.427/B.1.429, which has been associated with higher transmissibility and lower sensitivity to mAbs and vaccine sera [12], and other mutations/deletions in the NTD and RBD directly linked to increased resistance.

**Figure 2:**
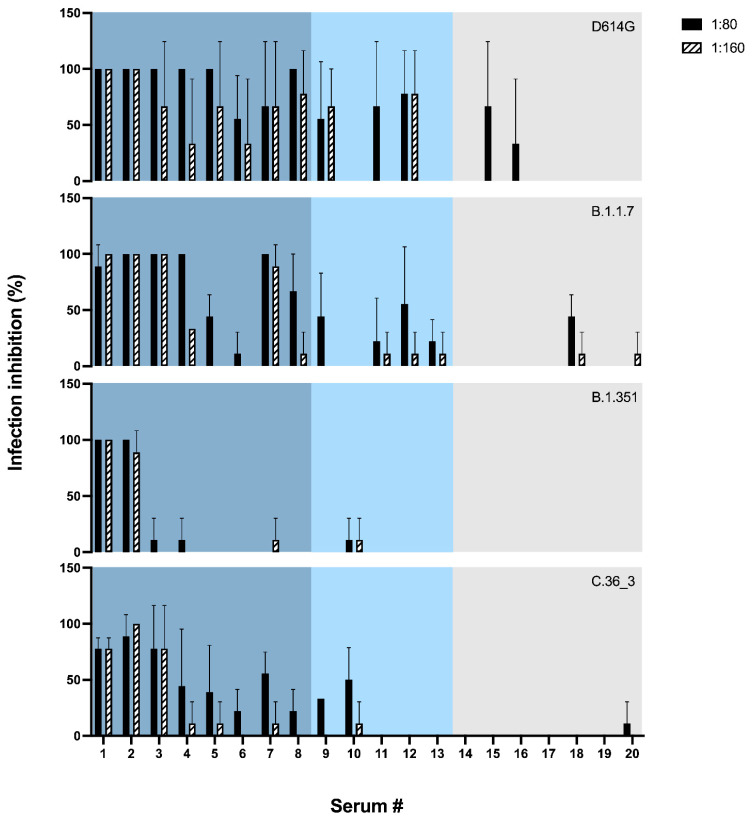
Neutralization assay. Neutralizing activity was assessed using two sera dilutions (1:80 and 1:160) against 0.01 MOI of four SARS-CoV-2 variants: D614G (black), B.1.1.7 (blue), B.1.351 (orange), C.36 (green). Mean values + SD are reported, each condition was tested in triplicate. Sera are ordered according to the anti-RBD titer and reported in blue, light blue and grey for titer >2500 U/mL, 2500–1000 U/mL or <1000 U/mL, respectively.

To test whether the sensitivity of hCoV-19/Italy/LOM-UnINSU/2021 was indeed altered, we tested the virus isolated from the same NPS used for WGS for its sensitivity in vitro against twenty sera (numbered from #1 to #20) collected from vaccinated subjects a month after receiving the second dose of Pfizer-BioNTech COVID-19 Vaccine BNT162b2 (Comirnaty^®^). Interestingly, two subjects (#1 and #2) had also undergone asymptomatic COVID-19 before vaccination. Electrochemiluminescence binding assay (ECLIA) was used for the in vitro quantitative determination of serum antibodies able to bind the RBD of the spike protein (Elecsys^®^ Anti-SARS-CoV-2 S, Roche). Anti-RBD and anti-S1/S2 titers are reported in Table S2. The neutralizing activity of these sera was also evaluated against three other SARS-CoV-2 strains isolated in our laboratory and belonging to the variants D614G, B.1.1.7 and B.1.351 (hCoV-19/Italy/LOM-UniSR-1/2020, clade G, Pango Lineage B.1; hCoV-19/Italy/LOM-UniSR7/2021, Clade GRY, Pango Lineage B.1.1.7; and hCoV-19/Italy/LOM-UniSR6/2021, Clade GH, Pango Lineage B.1.351.2). Results reported in Figure 2 show that all tested isolates have a sensitivity profile to serum neutralization proportional to the anti-RBD titer. The C.36_3 isolate has a profile comparable to B.1.1.7 for high anti-RBD titer sera but closer to B.1.351 for mid- and low-titer sera.

In conclusion, at the two tested sera dilutions, strain hCoV-19/Italy/LOM-UnINSU/2021 showed a reduced sensitivity profile the closest to VOC B.1.351 (Figure 2).

**Figure 3:**
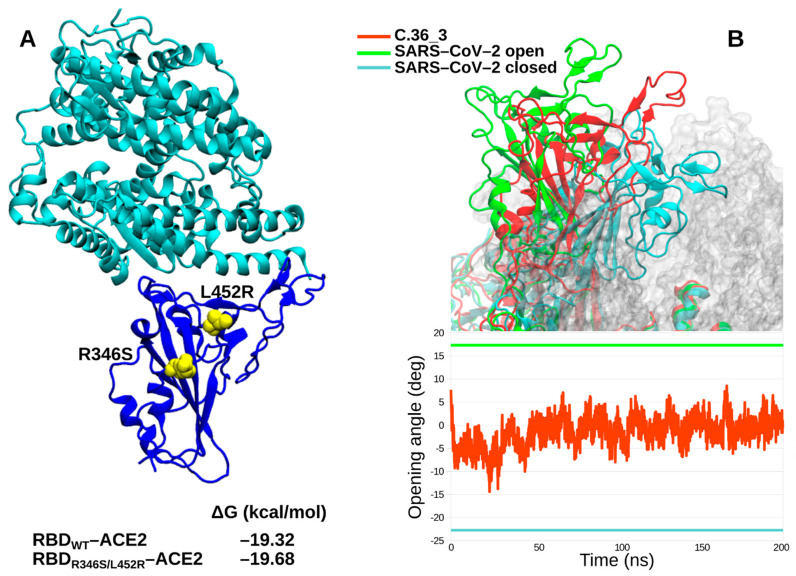
In silico spike characterization. (**A**) Structure of RBD-ACE2 complex with the relevant RBD mutations highlighted in yellow. The calculated affinity of wild type and mutated RBDs for ACE2 is reported. (**B**) C36_3 spike ectodomain representative conformation in the 1–up state (RBD colored in red), with SARS-CoV-2 1–up and closed RBDs highlighted in green and cyan, respectively (upper panel). C36_3 RBD opening angle over the MD simulation (lower panel, the three spikes follow the same coloring scheme).

We further characterized in silico the C.36_3 Spike in terms of its RBD affinity for ACE2 and its ectodomain dynamics. Affinity calculations did not show any obvious difference between the wild type RBD and the R346S/L452R double mutant (Figure 3A). Intriguingly, the spike dynamics showed a lower average exposure of the RBD compared to ancestral SARS-CoV-2 cryo-electron microscopy structures (Figure 3B). Therefore, from our simulations, we identified C.36_3 spike structural features compatible with a shift towards a more closed conformation that suggest a higher global protection of RBD neutralizing epitopes. At the same time, we do not envision a higher fitness due to these global features and an unaltered affinity for ACE2.

In summary, we report the preliminary characterization of a C.36_3 isolate carrying a highly mutated spike that was sequenced for the first time in Europe on 20 February 2021 and has experienced an increased circulation in Lombardy and Europe, albeit at low incidence. The spike mutational pattern causes a unique RBD exposure —as evaluated in silico— and a lower sensitivity profile to neutralization compared to the D614G variant, whose extent will require further investigation by sera titration assay. Moreover, during the manuscript preparation, we identified another C.36_3 isolate (hCoV-19/Italy/LOM-UniSR9/2021) in a direct contact of the patient infected with hCoV-19/Italy/LOM-UnINSU/2021. Whole-genome sequencing showed the same spike mutations and the alarming contextual emergence of E484K, one of the RBD mutations most frequently associated with decreased susceptibility per se, as well as in VOI B.1.526, VOC B.1.351, and P.1, and recently identified in the lineage of VOC B.1.1.7 [13]. While a more thorough characterization of current lineage C.36 isolates is needed and ongoing, our findings recommend the close monitoring of lineage C.36 diffusion and possibly its inclusion in the VOI list.

## Data Availability

SARS-CoV-2 whole-genome sequences of isolates reported and used in this study were deposited at the GISAID database under the following IDs: hCoV-19/Italy/LOM-UniSR-1/2020: EPI_ISL_413489, hCoV-19/Italy/LOM-UniSR7/2021: EPI_ISL_1924880, hCoV-19/Italy/LOM-UniSR6/2021: EPI_ISL_1599180, hCoV-19/Italy/LOM-UniSR9/2021: EPI_ISL_2023049 and hCoV-19/Italy/LOM-UnINSU/2021: EPI_ISL_1509923.

## Supplementary Materials

The following are available online at https://www.mdpi.com/article/10.3390/v13081514/s1. Table S1: Geographic distribution of the three C.36 sublineages expressed as the percentage of sequenced isolated by country over the total worldwide. Table S2: gender, age, anti-RBD titer and anti-S1/S2 titer of tested sera, colored according to the anti-RBD titer (blue, light blue and grey for titer >2500 U/mL, 2500–1000 U/mL or <1000 U/mL, respectively).
