# Supplementary material for: Characterization of a Lineage C.36 SARS-CoV-2 Isolate with Reduced Susceptibility to Neutralization Circulating in Lombardy, Italy
Source: Viruses. 2021 Jul 31;13(8):1514. doi: 10.3390/v13081514 (PMC8402759; doi:10.3390/v13081514)
Supplement: Supplementary file 1 [file viruses-13-01514-s001.zip › viruses-1282568-si final.pdf]

	Sublineage 1	Sublineage 2	Sublineage 3
**Australia**	0	4.6	0
**Canada**	0	5	0
**Denmark**	0	6.2	0
**Egypt**	26.2	26.1	0
**England**	22.4	17	29.7
**Germany**	18.6	7.1	10.1
**Italy**	2.9	0	10.1
**Lithuania**	2.9	0	0
**Switzerland**	4.8	4.6	7.6
**UAE**	0	5	0
**USA**	4.3	11.6	28.5
**Others**	18.1	12.9	13.9

Table S1: Geographic distribution of the three C.36 sublineages expressed as the percentage of sequenced isolated by country over the total worldwide.

Serum #	Gender	Age	Anti-RBD (U/mL)	Anti-S1/S2 (AU/mL)	
				IgG	IgM
1	M	27	> 2500	363	0.17
2	F	53	> 2500	> 400	0.17
3	F	59	> 2500	> 400	3.11
4	F	40	> 2500	> 400	0.54
5	M	52	> 2500	> 400	6.57
6	F	59	> 2500	202	1.43
7	F	64	> 2500	> 400	4.18
8	F	25	> 2500	322	0.67
9	F	58	1715	208	0.85
10	M	62	1467	248	1.42
11	M	65	1427	196	0.64
12	M	36	1357	211	0.39
13	M	57	1326	198	0.68
14	M	48	893	127	0.49
15	F	50	829	123	0.19
16	F	56	701	149	0.07
17	M	44	671	178	0.38
18	M	57	639	173	0.38
19	F	64	567	231	0.37
20	M	54	386	204	0.44

Table S2: gender, age, anti-RBD titer and anti-S1/S2 titer of tested sera, colored according to the anti-RBD titer (blue, light blue and grey for titer >2500 U/mL, 2500 - 1000 U/mL or <1000 U/mL, respectively).
